# A comparison of the efficacy of varenicline and bupropion and an evaluation of the effect of the medications in the context of the smoking cessation programme

**DOI:** 10.1186/s12971-017-0116-0

**Published:** 2017-02-01

**Authors:** Ali Ramazan Benli, Selman Erturhan, Muhammet Ali Oruc, Pinar Kalpakci, Didem Sunay, Yeltekin Demirel

**Affiliations:** 1 Department of Family Medicine, Karabuk University, Medical Faculty, 78000 Karabuk, Turkey; 2 Altinyayla State Hospital, Altinyayla, 58470 Sivas, Turkey; 3 Etimesgut State Hospital, Etimesgut, 06790 Ankara, Turkey; 4 Cekerek Primary Care Unit, Cekerek, 66500 Yozgat Turkey; 5 Department of Family Medicine, Cumhuriyet University, Medical Faculty, 58000 Sivas, Turkey

**Keywords:** Varenicline, Bupropion, Smoking cessation

## Abstract

**Background:**

Within the context of the support program for smoking cessation, initiated by the Turkish Ministry of Health in 2011, those who present at ‘smoking cessation’ centres and are found to be suitable for pharmacological treatment are given varenicline and bupropion free of charge. As the smoking cessation programme is centralized, the selection of the medication is made randomly to provide a fixed distribution rate. The aim of this study was to evaluate the efficacy of both varenicline and bupropion in smoking cessation and to evaluate the effect of the smoking cessation programme.

**Methods:**

A total of 405 individuals who met the study criteria were included in the study. Smoking habits and degree of dependence were determined in all the participants with the Fagerstrom test for nicotine dependence (FTND) and bupropion or varenicline therapy was initiated in those who were eligible. Patients were followed up at 15 days then at 1, 2, 3, 6 and 12 months after smoking cessation. A level of CO < 5 ppm and ‘point prevalence abstinence’ were used as the criteria of success for smoking cessation and this evaluation showed the non-smoking status in the previous 7 days.

**Results:**

The mean age of the participants was 35.19 ± 7.73 years and 82.8% (*n* = 334) were male. Of the participants, 60.2% (*n* = 244) were given varenicline and 39.8% (*n* = 161) bupropion. The mean FTND and package/year was not significantly different between the groups. The rates of success in the 1st and 2nd weeks, and 1st, 3rd and 6th months were significantly higher in the varenicline group than in the bupropion group (*p* < 0.05). At the end of one year, the rate of smoking cessation was determined as 13.9% (*n* = 34) in the varenicline group and 6.2% (*n* = 10) in the bupropion gruop. The difference was statistically significant (*p* = 0.015). At the end of 1 year when the previous 7 days smoking status was evaluated with the ‘point prevalence abstinence’ measurement as the success criteria, success rates were 20.5% with varenicline and 18.6% with bupropion and the difference was not significant (*p* = 0.646). The individuals who used the medications for 45 days or longer were more successful in smoking cessation (*p* < 0.001). The most common reasons given for discontinuing the medication were the side-effects (31.5%). No significant difference was determined between the groups in respect of the side-effects observed.

**Conclusions:**

Although the rates of smoking cessation in all the other control points were higher with varenicline than with bupropion, no significant difference was found between the success rates of varenicline and bupropion used in smoking cessation based on the last 7 days at the end of one year. Those who used the medications for 45 days or longer were more successful in smoking cessation.

## Background

Smoking is addictive in nature because of the psychoactive nicotine substance contained in tobacco. The risk of dependence in smoking is similar to that in heroin and alcohol use [1]. Smoking is one of the most important causes of four diseases which are the most common causes of mortality (atherosclerotic cardiovascular disease, cerebrovascular disease, cancer and chronic obstructive pulmonary disease) The development of smoking-related diseases and the associated risks of death significantly decrease in persons who quit smoking [2]. Therefore, one should seek help from both the immediate vicinity and professionals. Pharmacological support is an important component of smoking cessation and should be recommended to those want to quit [2–4].

Despite increased understanding of the harmful effects, smoking has recently become almost an epidemic. A new tobacco control period was initiated by the World Health Organization in 2003 within the context of action against smoking and the Framework Convention on Tobacco Control (FCTC) contract was published. Turkey signed that contract in 2004 and adopted it in the same year with Law no 5261. In accordance with that contract, the “National Tobacco Control Program” and the “Tobacco Control Action Plan” covering 2008–2012 were prepared with the participation of a large number of representatives from official and non-governmental organizations. Vareniclin tartrate (Champix ©) and bupropion hydrochloride (Zyban ©) which are used in smoking cessation were distributed free of charge in the smoking cessation clinics (SCC) for a 3-month period [5]. This increased applications to smoking cessation clinics. During this period, that there was an increase of 8.8 fold in the number of applications to our clinic was an encouraging finding in favour of the campaign. Patients who were to receive the medication were randomly determined by the medication support center in order to provide a constant distribution rate of varenicline and bupropion and so that physicians would not be aware of the medication distribution. This created an open experimental setup for comparison of the rate of success between varenicline and bupropion.

In this context, the aim of the study was to evaluate the efficacy of both varenicline and bupropion in the cessation of smoking and the effect of the smoking cessation programme. In addition, compliance of individuals to using the medications and side effects were evaluated.

## Methods

Our polyclinic has been in service since 1999, providing behavioural therapy support with a biopsychosocial approach to individuals wishing to stop smoking and recommending pharmacological methods and where possible monitoring the patients with proactive telephone calls and follow-up visits. Patients can apply to the clinic directly by calling the Ministry of Health ‘stop smoking’ helpline and making an appointment.

This study was designed as a prospective, comparative study. During the study period of March 2011-October 2011, a total of 2397 individuals aged over 18 years applied to our smoking cessation polyclinic and were included in the smoking cessation programme of the Ministry of Health. After smoking cessation, the participants were evaluated at 15 days then at 1, 2, 3, 6 and 12 months. Patients who did not attend the follow-up visits were called by telephone. A total of 405 individuals completed one year of follow-up and agreed to participate in the study.

A questionnaire was completed at face-to-face interviews. The questionnaire included sociodemographic characteristics of the participants and questions related to alcohol consumption (unit/day; 1 unit alcohol = 1glass of wine, 1 bottle of beer, 1 measure of spirits), smoking status since stopping, medication use and if used irregularly, the reasons why (patients were not guided in this), side-effects, confidence in their ability to be successful in smoking cessation (0–5 points;0: no, 1: very low, 2: low, 3:medium; 4:high, 5:very high), whether or not there was another smoker at home, support from family and friends and previous attempts to stop smoking. In addition to the physical examination, the Fagerstrom Test for Nicotine Dependence (FTND) was applied [6].

In the period of medication support, the medicines to be used by patients specifically in this period were distributed free of charge by the Ministry of Health. Persons considered eligible after presentation were given one of the two medications (varenicline or bupropion) by the support center. Since nicotine replacement was not included in the program, this study only evaluated the bupropion and vareniclin therapies. The participants were informed about the frequent possible side effects of the medications. Of the total participants, 60.2% (*n* = 244) were given vareniclin and 39.8% (*n* = 161) bupropion. Patients receiving treatment for previously known psychiatric disease and those with epilepsy were excluded from the study.

### Fagerstrom Test for Nicotine Dependence (FTND)

FTND is a widely used scale in the determination of nicotine dependence. The Turkish version of FTND has been tested for reliability. FTND consists of 6 questions scored between 1 and 10 points with a score of > 4 points indicating the possibility of dependence. Based on the total score obtained from a comprehensive evaluation of this test; nicotine dependence is graded in 5 categories as very low (0–2 points), low (3–4 points), medium (5 points), high (6–7 points) and very high (8–10 points) [6].

### Evaluation of side effects

Questions were asked about the possible side effects to achieve a standard conclusion and to learn if there were side effects that the patients could not recall, but might have experienced. In addition, other side effects reported by the patients were recorded and the side effects specified by the patients were combined if they were very similar.

### Measurement of CO level in breath

The monitoring of smoking status was implemented by measuring the CO level in the breath using a ‘Bedfontpico + Smokerlyzer’ monitor in the clinic at every visit. In the CO measurement in the breath, participants were first asked to perform a forced expiration and then a full inhalation following by holding their breath until a beep sound from the device (about 15 s) and then to blow slowly into the mouthpiece, aiming to empty the lungs completely. For subjects who reported that they had not smoked in the previous 7 days, the CO measurements were performed twice at 5 min intervals. A level between 6 and 10 ppm indicated an infrequent smoker or danger zone, while 11 ppm and higher values indicated an addicted smoker with high levels of CO in their blood. In the present study, a CO level ≤5 ppm was considered as the success criterion as proposed by Perkins et al. [7].

The ‘point prevalence abstinence’ measurement, as in the American Public Health Service guidelines, was used to determine the success rate in smoking cessation [8]. This evaluation expresses the smoking status in the previous 7 days and has been widely used in previous studies as it provides a general result and increases statistical power [9].

### Statistical analysis

Statistical analysis of the data was performed utilizing the SPSS v20.0 Statistical Software. Definitive statistics were expressed as mean rank and difference for continuously measured variables and as frequency and percentage for nominal variables. Relationships between variables were evaluated using correlation analysis. The Chi-square test was used for categorical variables, while variables specified with measurement were evaluated through the Independent Samples *t*-test. A value of *p* < 0.05 was considered statistically significant.

Approval for this study was granted by the Local Ethics Committee.

## Results

Of the total 405 participants, 82.5% (*n* = 334) were male and 17.5% (*n* = 71) female. The age of the patients ranged from 20 to 65 years. The overall mean age was 35.24 ± 7.7 years, while the mean age was 35.09 ± 7.85 years in males and 36.03 ± 6.88 years in females (Table 1). The mean FTND value at presentation was determined as 6.34 ± 2.38 for varenicline and 6.23 ± 2.49 for bupropion (*p* = 0.655), while package/year values were 14.8 ± 7 and 15.9 ± 6, respectively (*p* = 0.624).

**Table 1:** The demographical characteristics and smoking habits of participants

Variable		Varenicline (*n* = 244)		Bupropion (*n* = 161)		p*
		n	%	n	%	
Gender	Male (*n* = 334)	194	79.5	140	87.0	0.054
	Female (*n* = 71)	50	20.5	21	13.0	
Age Groups	20–29 (*n* = 111)	74	30.3	37	23.0	0.267
	30–39 (*n* = 184)	106	43.4	78	48.4	
	≥40 (*n* = 110)	64	26.2	46	28.6	
Marital Status	Married (*n* = 329)	191	78.3	138	85.7	0.061
	Not Married (*n* = 76)	53	21.7	23	14.3	
Educational Status	Primary- Secondary	45	18.4	31	19.3	0.775
	High School	101	41.4	71	44.1	
	College + ^a^	98	40.2	59	36.6	
Alcohol Consumption Status	Yes (*n* = 63)	37	15.2	26	16.163	0.789
	No (*n* = 342)	207	84.8	135	83.9	
Social support	Yes (*n* = 241)	169	69.3	75	65.2	0.394
	No (*n* = 161)	75	30.7	86	34.8	
The amount of cigarettes smoked per day in the initial presentation	1–10 pcs	29	11.9	5	3.1	0.004
	11–20 pcs	99	40.6	86	53.4	
	21–30 pcs	79	53.4	43	26.7	
	≥31 pcs	37	45.7	27	16.8	
Previous quitting trial	Yes (*n* = 272)	167	68.4	105	65.2	0.499
	No (*n* = 133)	77	31.6	56	34.8	
Concerns about the harmful effects of smoking	No (*n* = 24)	16	6.6	8	5.0	0.532
	Little (*n* = 69)	45	18.4	24	14.9	
	High (*n* = 170)	96	39.3	74	46.0	
	Very High (*n* = 142)	87	35.7	55	34.2	
Mean Fagerstrom Score		6.34 ± 2.38		6.23 ± 2.49		0.655**
The amount of cigarettes/day in the initial presentation		22.78 ± 10.11		23.39 ± 9.13		0.540
Mean Age		34.8 ± 7.61		35.9 ± 7.78		0.124
Mean age of start to begin smoking		17.80 ± 4.26		17.60 ± 4.35		0.635
Mean Body Mass Index		25.46 ± 3.80		26.11 ± 3.59		0.082

*Chi square test, **Independent sample *t* test (in evaluation of mean values)

^a^College graduate or bachelor’s degree

At the end of one year, the success rate was found to be 20.5% with varenicline and 18.6% with bupropion based on 7-day point prevalence (*p* = 0.646). In persons who used the medications for 45 days or longer, the one-year success rates were determined as 27.6 and 27.8% for varenicline and bupropion based on 7-day point prevalence (*p* = 0.987). The CO levels of all the participants were measured and found to be <5 ppm in 80 participants who stated that they had not smoked during the previous week (mean CO level, 2.36 ± 0.90).

Of the total participants, 3.95% (*n* = 16) never used the medication, while 6.7% (*n* = 27) used the medication regularly. Regular use of the medication over 90 days was determined in 9.8% (*n* = 24) of the varenicline users and 1.9% (*n* = 3) of the bupropion users (*p* = 0.002). Medication use of 71 days or longer was determined in 34 (13.7%) varenicline and 6 (3.7%) bupropion users (*p* = 0.001). The rate of medication use for at least 45 days was determined as 25.9% (*n* = 105) (varenicline; *n* = 87, bupropion; *n* = 18) (*p* < 0.001).

The demographic characteristics and smoking habits of the participants are shown in Table 1. No significant differences were observed between the groups in respect of gender, age, marital status, educational level, BMI, presence of another smoker at home, alcohol consumption, social support, previous quitting attempts, concerns about the harmful effects of smoking, Fagerstrom scores or the age of starting to smoke. However, there was a significant difference between the groups in terms of the amount of smoking, with a higher rate determined in the varenicline group than in the bupropion group (*p* = 0.004).

Based on the patient self, the period with the highest rate of success was the first two weeks after the day of cessation. The rates of success in the first and second weeks were significantly different in favour of varenicline (*p* < 0.001). The rate of success in the first month was determined as 48.4% in varenicline and 33.5% in bupropion users (*p* = 0.003). When the subjects who did not smoke in the first 3 months were evaluated, the varenicline users were found to be significantly more successful than the bupropion users (*p* = 0.003). The difference between the two groups continued at the end of 6 months and at the end of one year, the rate of the smoking cessation was found to be 13.9% (*n* = 34) in the varenicline group and 6.2% (*n* = 10) in the bupropion group with a statistically significant difference (*p* = 0.015) (Table 2).

**Table 2:** Comparisons of smoking cessation of medication groups in follow-up

Period	Varaenicline (*n* = 244)		Bupropion (*n* = 161)		Total (*n* = 405)		p*
	n	%	n	%	n	%	
First week	158	64.8	75	46.6	233	57.5	<0.001
First 2 weeks	143	58.6	63	39.1	206	50.9	<0.001
First 1 month	118	48.4	54	33.5	172	42.5	0.003
First 3 months	76	31.1	29	18.0	105	25.9	0.003
First 6 months	48	19.7	16	9.9	64	15.8	0.009
First 1 year	34	13.9	10	6.2	44	10.9	0.015

*Chi-square test

Open-ended questions were asked about the reasons for taking the medication, but not using it regularly and the answers given were categorized. Those who participated in the campaign because it was free of charge although they did not feel ready to quit smoking or did not believe that the medication would be effective were determined at the rate of 10.1%. After exclusion of 27 individuals who used the medications regularly, the final assessment was applied to 378 participants. The first or the most remarkable reasons reported by the participants are given in Table 3 in order of frequency. According to this, 30% of those using varenicline and 33.5% of those using bupropion, stated side effects as the reason for not using the medication regularly. With the exception of 16 subjects who never used the medications, 62.2% (*n* = 242) of the remaining 389 individuals reported that they developed at least one side effect. Of the total 234 varenicline users, 143 (61.1%) reported that they experienced at least one side effect, while this rate was 98 (63.2%) of 155 bupropion users (*p* = 0.674). The 5 most common side effects were nausea, insomnia, headache, fatigue, irritability and the 5 side effects most commonly self-reported in the study were insomnia, nausea, irritability, headache and dry mouth.

**Table 3:** Comparisons of causes of irregular medication usage of groups

The cause of irregular medication usageuse	Varenicline (*n* = 220)		Bupropion (*n* = 158)		P*
	n	%	n	%	
Side effects	66	30	53	33.5	0.464
Over self-confidence/no more need for medicationthe drug	48	21.8	17	10.8	0.005
Not to see any effect of the drugmedication	14	6.4	21	13.3	0.022
Not feel exactly ready to quit smoking	14	6.4	16	10.1	0.182
Fail to quit smoking	18	8.2	6	3.8	0.085
Begin to smoke again	14	6.4	8	5.1	0.594
Fear of side effects	10	4.5	6	3.8	0.722
Inability to come controls	7	3.2	7	4.4	0.526
Not to believe effect of the medicationdrug	6	2.7	5	3.2	0.803
Increased in desiremand to smoke	4	1.8	4	2.5	0.635
ThinkDesire to quit the to not to take the medication anddrug smoking at the same dday of smoking cessation	3	1.4	4	2.5	0.406
Work schedule	3	1.4	1	0.6	0.493
Since the drug is bupropion	0	0	4	2.5	0.018
ToNot dis like using medicationine	2	0.9	0	0	0.229
Not to take the drug with them	1	0.5	2	1.3	0.381
Noncompliant	10	4.5	4	2.5	0.307

*Chi square test

When the factors affecting the success of smoking cessation were evaluated in 80 participants who quit smoking, the rate of success was higher among those who did not drink alcohol, used the medication for 45 days or longer or had experienced multiple failed attempts to quit smoking (*p* < 0.05). The cessation of smoking rate in the previous 7 days of those who used the medications for 45 days or more was 27.6% in the varenicline group and 27.8% in the bupropion group. No significant differences were observed between the groups in respect of other parameters (Table 4).

**Table 4:** Parameters that considered to effect quitting success

Parameters		n	%	*P* ^*^
Gender	Male (*n* = 334)	61	18.3	0.102
	Female (*n* = 71)	19	26.8	
Age	20–29 (*n* = 111)	28	25.2	0.234
	30–39 (*n* = 184)	33	17.9	
	≥40 (*n* = 110)	19	17.3	
Marital status	Married (*n* = 329)	64	19.5	0.752
	Not married (*n* = 76)	16	21.1	
Education	Primary, secondary, high school (*n* = 248)	42	16.9	0.804
	University (*n* = 157)	38	24.2	
Alcohol consumption	Yes (*n* = 63)	5	7.9	0.01
	No (*n* = 342)	75	21.9	
Social support	Yes (*n* = 274)	50	18.2	0.271
	No (*n* = 131)	30	22.9	
Previous quitting attempts	≤1 time (*n* = 133)	41	15.5	0.003
	>1 time (*n* = 272)	39	27.7	
Concern about harmful effects of smoking	No (*n* = 24)	8	33.3	0.028
	Little (*n* = 69)	19	27.5	
	High (*n* = 170)	34	20.0	
	Very high (*n* = 142)	19	13.4	
Receiving medication	≥45 days (*n* = 105)	29	27.6	0.019
	<45 days (*n* = 300)	51	17.0	
Self-confidence about quitting	0–4 points (*n* = 303)	52	17.2	0.024
	5 points (*n* = 102)	28	27.5	
Another person smoking in house	Yes (*n* = 142)	29	20.4	0.804
	No (*n* = 263)	51	19.4	

*Chi square test

## Dıscussıon

In this study, the overall success rate of both medications in smoking cessation was found to be 19.8% at the end of the first year. Based on the patient statements, the uninterrupted rate of cessation (URC) of varenicline was found to be higher in the first month, in the first three months, 5–12 weeks, in the first 6 months and the entire year. At the end of one year, the rate of smoking cessation was determined as 13.9% in the varenicline group and 6.2% in the bupropion group. However, when the ‘point prevalence abstinence’ measurement of the American Public Health Service guidelines, was used as the criteria of success in smoking cessation in the previous 7 days at the end of 1 year, the rate of success was determined as 20.5% for varenicline and 18.6% for bupropion. No statistically significant difference was found between the medications.

In a systematic review by Cahill et al., varenicline was superior to single forms of NRT (OR 1.57; 95% CI, 1.29 to 1.91), and to bupropion (OR 1.59; 95% CI, 1.29 to 1.96), but was not more effective than combination NRT (OR 1.06; 95% CI, 0.75 to 1.48) [10]. However, in that review, the outcome for benefit was continuous or prolonged abstinence for at least six months from the start of treatment [11]. In meta-analyses by Hughes et al. [12] good results were found to be related to varenicline compared to bupropion. In another systematic review which examined 10 studies, findings suggested that varenicline groups achieved higher rates of abstinence compared to both NRT and placebo, bupropion and NRT were of similar effectiveness, and bupropion and varenicline both had higher abstinence rates compared to placebo [13]. No statistically significant difference in terms of side effects was seen in either medication.

CO measurement is frequently used to confirm smoking cessation. In a recent study, a CO titer of 5 ppm was stated to be the most optimal value containing both sensitivity and specificity [7]. Likewise in the present study, a CO level ≤5 ppm was accepted as the criterion of success. In the current study, the rate of success based on 7-day point prevalence was found to be 20.5 and 18.6% for varenicline and bupropion, respectively. The difference was not statistically significant. In literature, 1-year rates of success based on 7-day point prevalence have been reported as 26.2 and 30.5% for varenicline and between 20.7 and 35% for bupropion [14–16].

In previous studies about smoking cessation, the mean age has ranged between 37.8 and 46.2 years [17–19]. Although there have been national and international studies reporting that age does not influence smoking cessation outcomes [17, 20], some studies have found that an older age has a positive influence on the success of smoking cessation [12, 14–21]. The relatively lower mean age in the current study may be a factor in the lower success rate of cessation.

In this study, the rate of regular medication use over 3 months was 6.7%. In a previous study evaluating the use of varenicline therapy, 28.2% of the participants continued the therapy for 3 months. In the same study, the rate of success was significantly higher in the group which received therapy for 3 months or longer [22]. In a study by Sheffer et al., the rate of completion of 3-month therapy was 40% and the successful use of the therapy was found to be associated with maintaining communication [23]. Proactive calls have been shown to increase the rate of success [24]. Low compliance to treatment is remarkable in the current study. This might have resulted from the great number of submissions in the campaign period and the lack of proactive calls. In the current study, the rate of success was significantly higher in those who used the medication for longer than 45 days. In a study by Stapleton et al., the 6-month uninterrupted success rate of participants who completed the therapy was determined as 37.9%, while this rate was only 15.6% in those who discontinued the medication early. In this respect, it can be said that the current study is in parallel with literature [25].

When the subjects who used the medications for 45 days or longer were evaluated, the one-year success rate based on 7-day point prevalence was similar between the groups. In literature, the one-year success rate based on 7-day point prevalence has been reported as 26.2–30.5% for varenicline and 20.7–35% for bupropion. The results of the current study are consistent with literature in this respect [18, 20, 21].

In the current study, 31.5% of the participants reported that they stopped using the medications because of side effects. No statistically significant difference was observed between the two medications in terms of side effects and quitting the medication because of side effects. In a systematic review by Lei et al. [11] discontinuation because of adverse events (RR, 1.34;95% CI, 1.02–1.75) was significantly more common for the non-NRT group, which experienced more serious adverse events (RR, 1.87; 95% CI, 1.08–3.24), compared with the control group. As NRT was not used in the current study, a comparison could not be made. Other reasons for not using the medications regularly were lack of confidence in oneself and belief that there was no further need of treatment (21.8%) in the varenicline group and not seeing any benefit from the medication use (13.3%) in the bupropion group. However, the rate of persons who joined the campaign because it was free of charge although they did not feel ready to quit smoking or did not believe that the medication would be effective was 10.1%. In a previous study, the most important reason for discontinuation of the medication was starting smoking again (41.6%) [26]. Other common causes included side effects of the medication and the belief that no further treatment was required. The rate of smoking has been reported to be high among alcohol and substance abusers which makes smoking cessation difficult [27]. In the current study, smoking cessation was achieved by 5 (7.9%) alcohol abusers and 21.9% of individuals who did not drink alcohol and the difference was statistically significant.

It has been reported in literature that those who are confident in their ability to quit smoking are more successful [28]. Consistent with the literature, subjects in the current study with self-confidence about smoking cessation were found to be more successful. According to Prochaska [29] and Hymowitz [30], the more attempts that are made to quit smoking, the higher the rate of success will be. Similarly in the current study, the difference in the rate of success was statistically significant in respect of the number of attempts to quit smoking.

Although the results of this study are in parallel with reports in literature, there were some limitations to the study. The sudden increase in patient numbers due to the campaign and not being able to make sufficient proactive follow-up calls for continuous monitoring can be considered to have seriously reduced the success rates. Despite giving the patients appointment cards for the follow-up visits and emphasising the importance of those monitoring visits, the rate of those attending follow-up visits remained low. The significant factors for this situation were found to be the over-subscription of patients in the campaign period, a single doctor was attending a large number of patients and that proactive follow-up telephone calls could not be made. Previous studies have shown that proactive calls in the support of smoking cessation were related to an increase in success rates [24, 31, 32].

## Conclusıon

At the end of the first year, no significant difference was found between the medications in terms of the success of smoking cessation. The rate of success of both the medications was consistent with literature. Persons who used the medications for longer than 45 days were more successful than those used them for shorter periods. In this study, the rate of compliance to treatment was lower compared to previous reports in literature. This may be attributable to the participation of the individuals without fully matured thoughts of quitting, purely because of the free-of charge distribution of the medications and also the low mean age of the participants. The lack of proactive calls might also have been a factor affecting compliance. The free of charge smoking cessation program launched by the Ministry of Health has created awareness. Although no significant difference was observed between the medications in terms of smoking cessation, the very low compliance to treatment suggests that such campaigns should be re-organized using different strategies.

## Abbrevıatıons

FTND: Fagerstrom test for nicotine dependence
NRT: Nicotine replacement therapy
